# Spectroscopic Analysis of an Antimalarial Drug’s (Quinine) Influence on Human Serum Albumin Reduction and Antioxidant Potential

**DOI:** 10.3390/molecules27186027

**Published:** 2022-09-15

**Authors:** Wojciech Rogóż, Olga Lemańska, Jadwiga Pożycka, Aleksandra Owczarzy, Karolina Kulig, Tammam Muhammetoglu, Małgorzata Maciążek-Jurczyk

**Affiliations:** 1 Department of Physical Pharmacy, Faculty of Pharmaceutical Sciences in Sosnowiec, Medical University of Silesia in Katowice, 40-055 Katowice, Poland; 2 Diagnostyka S.A., 42-202 Częstochowa, Poland

**Keywords:** quinine, human serum albumin, antioxidant and reduction potential, spectroscopy

## Abstract

Quinine (Qi) is a well-known drug used in malaria therapy; it is also a potential anti-arrhythmic drug used in the treatment of calf cramps, rheumatoid arthritis, colds, and photodermatitis. Moreover, it is used in the food industry for the production of tonics. This study aimed to analyze the interaction between quinine and a transporting protein—human serum albumin (HSA)—as well as the influence of Qi on both protein reduction and antioxidant potential. It was found that Qi (via spectrofluorometric measurements and circular dichroism spectroscopy) binds to HSA with a low affinity and slightly affects the secondary structure of albumin. As demonstrated by the use of ABTS and FRAP assays, HSA has a higher antioxidant and reduction potential than Qi, while their mutual interaction results in a synergistic effect in antioxidant activity and reduction potential.

## 1. Introduction

Quinine (Qi) and its derivatives, such as chloroquine and primaquine, belong to the oldest effective antimalarial drugs. The antimalarial activities of these substances result from the quinoline scaffold construction [1,2]. Qi is an alkaloid derived from the bark of the South American cinchona tree [3,4]. Although Qi has a long history, and other new antimalarial drugs are available, this substance is still used in medicine. It shows high efficiency and is sometimes used with other medications, such as doxycycline [5]. Qi used during therapy may affect the endogenous antioxidant mechanism [6]. The level of free radicals in the human body has a large impact on the course of a disease. When the parasites from the Plasmodium species are released into the body of patients with malaria, red blood cells rupture. This process is accompanied by the growth in the level of oxygen radicals. It is one of the body’s key defense mechanisms [7]. The most important sources of free radicals in the human body are phagocytic cells, such as neutrophils and monocytes [8]. Endogenous and exogenous antioxidant mechanisms take part in regulating the levels of free radicals in the organism. These mechanisms are necessary because pathologically-high levels of free radicals in the human body have destructive effects on micro- and macromolecules, cells, and tissues [9,10].

Albumin is the most widespread protein in the blood and the most important in the human body. This protein transports various substances, such as drugs, and scavenges free radicals [11]. Albumin transports Qi and, thus, influences the course of malaria as well as the process of its treatment [12]. There are two main drug-binding sites (Sudlow sites I and II) located in albumin subdomains IIA and IIIA, respectively. The main aim of this study was to analyze the interaction between Qi and the transporting protein—human serum albumin (HSA)—as well as the influence of the binding on albumin’s antioxidant and reducing activity. Many antioxidants mutually alter their antioxidant potential. Qi binding by albumin and antioxidant activity were also analyzed [13,14,15]. HSA can participate in the regulation of the level of free radicals. The amino acid residues that are mainly responsible for the regulatory mechanism are Cys-34, Met-87, Met-123, Met-298, Met-329, Met-446, and Met-548, but to a small extent [16,17,18,19]. Under the influence of the reactive forms of oxygen, changes in the drug-binding ability are possible [20]. This phenomenon has to be taken into account because substances with high antioxidant potential (such as ascorbic acid (AA), α-tocopherol, and proanthocyanidins) bind with HSA [21]. The antioxidant activity of HSA is also strongly dependent on the environment’s pH and is probably connected with conformational changes in protein particles [22,23].

The analysis of antioxidants and reducing the potential of drugs (and drug influences) on the ability to scavenge free radicals by internal structures are very significant, and it allows us to better understand their effects on the body.

## 2. Results and Discussion

A high level of free radicals can lead to tissue damage and degradation while the formation of free radicals is a very important mechanism of body defense [24]. Due to these facts, an analysis of the antioxidant activity of pure Qi as well as an analysis of Qi binding with HSA was carried out [23,24,25,26,27,28,29]. In the first part of the study, the interaction between Qi and HSA at the molecular level using spectrophotometric and spectrofluorimetric methods was conducted, while in the second part of this work, the influence of Qi on the reduction and antioxidant activity of HSA was analyzed.

### 2.1. The Spectroscopic Analysis of Qi-HSA Interaction

**Figure 1:**
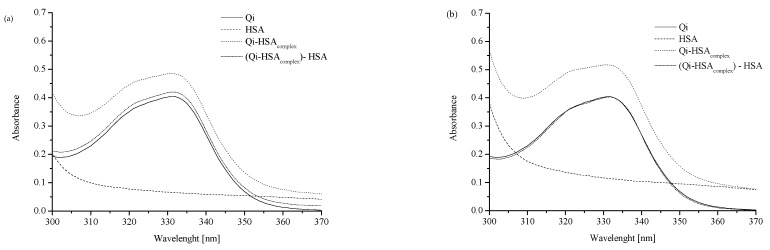

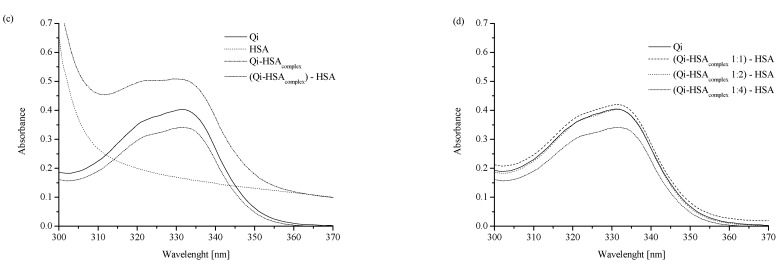
Absorption spectra of analyzed ligand–protein mixtures: (**a**) “Qi”, “HSA”, “Qi-HSA_complex_” and “Qi_calc_ = Qi-HSA_complex_ − HSA” at ligand:protein 1:1 molar ratio; (**b**) “Qi”, “HSA”, “Qi-HSA_complex_” and “Qi_calc_ = Qi-HSA_complex_ − HSA” at ligand:protein 1:2 molar ratio; (**c**) “Qi”, “HSA”, “Qi-HSA_complex_” and “Qi_calc_ = Qi-HSA_complex_ − HSA” at ligand:protein 1:4 molar ratio; (**d**) “Qi”, “Qi_calc_ 1:1 molar ratio”, “Qi_calc_ 1:2 molar ratio” and “Qi_calc_ 1:4 molar ratio”; [Qi] 7 × 10^−5^ M, [HSA] 7 × 10^−5^ M, 1.4 × 10^−4^ M, 2.8 × 10^−4^ M; all tested solutions were prepared in phosphate buffer (pH 7.4).

To analyze the interaction between Qi and HSA, the absorption measurements of HSA, Qi, and Qi in the presence of HSA were performed using UV-Vis spectroscopy. Different values of Qi:HSA molar ratios (1:1; 1:2, and 1:4) were used. Figure 1 presents absorption spectra of “Qi,” “HSA,” “Qi-HSA_complex_” (at Qi:HSA molar ratios 1:1, 1:2, and 1:4) as well as “Qi-HSA_complex_ − HSA” as a result of the mathematical subtraction “HSA” absorption spectrum from the “Qi-HSA_complex_” absorption spectrum.

The absorption spectra of Qi, HSA, and Qi-HSA_complex_ were analyzed in the wavelength range from 300 to 370 nm (Figure 1), and one significant Qi signal with maximum absorption at *λ_max_* 331 nm was observed. All analyzed solutions were prepared in phosphate buffer (pH = 7.4). With the increase in HSA concentration, it was observed that the Qi absorption spectrum changed (Figure 1). The values of the mean absorbance for “Qi” and “Qi_calc_ = Qi-HSA_complex_ − HSA” at *λ_max_* 331 nm in Qi-HSA_complex_ 1:1, 1:2, and 1:4 molar ratio were presented. If at the specific wavelength the values of two different substance absorbances differed from the absorbance of the sum of both components in the mixture, it probably meant that both substances could interact with each other. In the case of the absence of interaction between Qi and HSA, the absorption spectra for Qi, both measured and calculated as the HSA subtraction from Qi-HSA_complex_ (Qi_calc_ = Qi-HSA_complex_ − HSA), should overlap [30,31]. Regardless of the ligand:protein molar ratio, a difference in the values at *λ* = 331 nm between the Qi absorbance measured and calculated was observed (Figure 1). It probably confirms the formation of non-covalent bonds between the functional groups of Qi and amino acid residues of HSA or the occurrence of interaction between Qi and HSA. The greatest difference in the absorption value between Qi measured and calculated was observed when the molar ratio Qi:HSA was 1:4 (Figure 1c,d; Table 1). 

The analysis of the possibility of the protein–ligand interaction using UV-Vis spectrophotometry was also carried out by Suhartono et al. [31]. They analyzed the absorbance of BSA in the wavelength range from 220 to 300 nm, as well as samples of BSA with Cd^2+^ ions at an increasing concentration (BSA + 0.001 ppm of Cd^2+^; BSA + 0.01 ppm of Cd^2+^, and BSA + 0.1 ppm of Cd^2+^). The results of their research confirm that the use of UV-Vis spectroscopy allows for the detection of interactions between radiation and absorbing substances (e.g., the protein and ligand) and changes the protein structures under the influence of ligands [31]. Both of the above literature examples confirm the validity of the use of UV-Vis spectroscopy in this work. 

**Figure 2:**
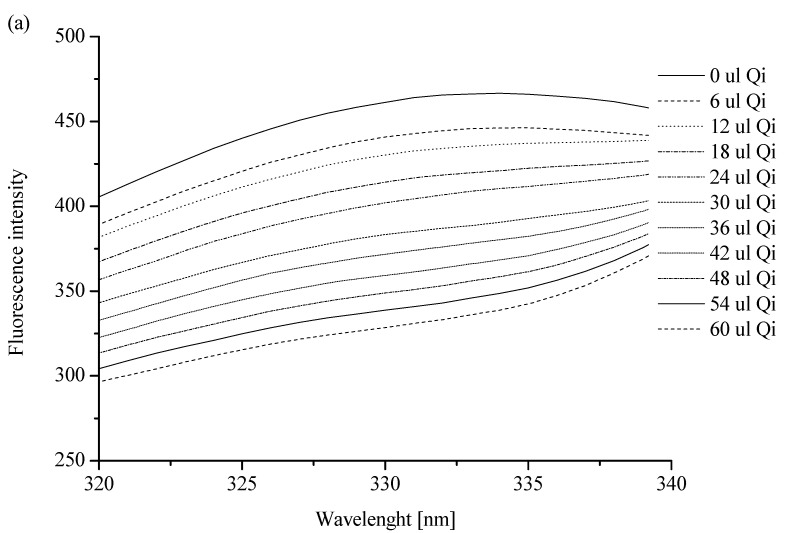

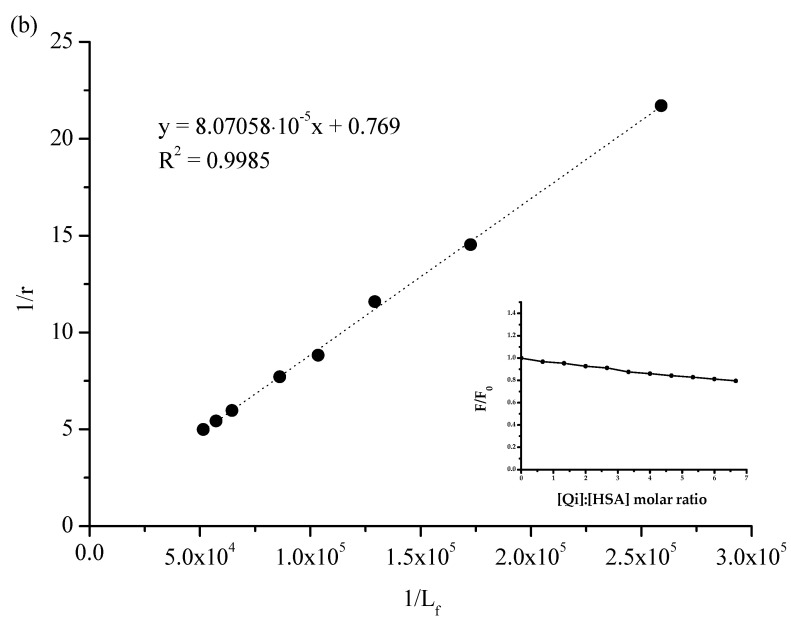
Spectrofluorimetric analysis: (**a**) the fluorescent emission spectra of HSA ([HSA] 3 × 10 ^−6^ M): without or in the presence of Qi ([Qi] 1 × 10^−3^ M); λ_ex_ 285 [nm]; (**b**) the Klotz plot for HSA in the presence of Qi at Qi:HSA molar ratio from 0.67:1 to 6.67:1. In the insert: quenching of HSA fluorescence by Qi at Qi:HSA molar ratio from 0.67:1 to 6.67:1; λ_ex_ 285 nm; [HSA] 3 × 10^−6^ M; [Qi] 1 × 10^−3^ M; pH = 7.4.

To confirm the interaction between Qi and HSA, spectrofluorometric measurements were performed. All analyzed solutions were prepared in phosphate buffer (pH = 7.4). The HSA solution was titrated by the Qi solution (*λ_ex_* 285 nm; pH = 7.4) and non-significant changes in albumin fluorescence intensity (Figure 2a) confirmed weak Qi-HSA binding. Similarly, in previous works by Wanwimolruk et al. [12] and Frostell-Karlsson et al. [32], low-affinity HSA binding sites for Qi were observed, while on the structures of other human serum proteins, such as alpha 1-acid glycoprotein, high-affinity binding sites for Qi were observed [12,33,34]. In a similar manner to our work, for the analysis of the stereoselective interaction of Qi and quinidine with bovine serum albumin (BSA), Liu et al. used the fluorometric method [35]. Based on the obtained data and the Klotz equation (Equation (2)), the Klotz curve (Figure 2b), the association constant (*K_a_*), and the number of Qi molecules bound to HSA (*n*) were determined (Table 2).

The association constant (*K_a_*) of Qi binding with albumin is relatively low and is equal to (0.952 ± 0.178) × 10^4^ M^−1^ (Table 2). Similar results were obtained by Frostell-Karlsson et al. [32] and Liu et al. [35]. Using isothermal titration calorimetry, Liu et al. gained the association constant of Qi and quinidine binding with BSA equal to (0.99 ± 0.03) × 10^4^ M^−1^, and this value did not change significantly with the temperature increase, from 288 to 308 K. Karlsson et al., in turn, observed that the value of *K_a_* of Qi binding with HSA equaled 2 × 10^3^ M^−1^. This result was obtained as a consequence of the use of surface plasmon resonance technology [32]. In this paper, the value of the association constant is connected with specific limitations of the applied method (the need to immobilize HSA). As Day et al. showed, Qi affinity is low for HSA as well as for serum albumin from other species (e.g., monkey, dog, donkey, pig, sheep, rat, goat, hamster, turkey, or chicken) [36]. Similarly, according to Kratochwil et al. [37] and Liu et al. [35], the number of drug molecules was almost 1, which pointed to one class of the HSA binding site for Qi (1.336 ± 0.274; Table 2). The value of the parameter n in the temperature range from 288 to 308 K did not change [35]. It is well known that a single HSA molecule is characterized by two specific drug binding sites, called Sudlow sites I and II [38], and taking into account the value of the association constant, a main Qi binding site was not likely located near these sites [12]. According to Kratochwil et al. [37] and based on the data obtained by Paubel et al. [39], although the value of the n parameter is 1, Qi does not bind both sites in subdomains IIA and IIIA [37,39].

Regardless of Qi’s weak binding with HSA, the changes in a protein’s tertiary and secondary structure are likely and, therefore, for the study of protein structures, circular dichroism (CD) spectroscopy was used. CD is an excellent method for the rapid evaluation of the secondary and tertiary structures as well as the folding and binding properties of proteins [40]. Proteins such as HSA possess a number of chromophores that give rise to CD signals. In the far UV region (180–240 nm), which corresponds to peptide bond absorption, the CD spectrum can be analyzed; for example, a strong double minimum (209 nm and 222 nm; characteristics for proteins with the dominance of α-helix in the secondary structure) or a single negative band (210–225 nm; characteristics for proteins with the dominance of a β-sheet in the secondary structure) may be observed. The CD spectrum in the near UV region (260–320 nm) reflects the environments of the aromatic amino acid side chains and, thus, gives information about the tertiary structure of the protein [41,42,43]. 

Due to the presence of some factors, e.g., ligands, changes in the structure of the protein are possible. Based on Equation (4), the values of mean residue ellipticity [Θ]_mrw_ for HSA, in both the absence and presence of Qi, were calculated and collected in Table 3. 

The data collected in Table 3 confirm that HSA is a protein with the dominant α-helix structure in its secondary structure. Similarly, as in the literature, the CD spectrum was characterized by two negative bands at *λ* 209 and 220.8 nm (Table 3) [44,45,46]; no changes in ellipticity (deg) at *λ* 200 and 250 nm led to the conclusion that the samples were prepared properly and the HSA concentration in both samples was the same [46]. Based on the standard deviation analysis, it can be concluded that significant changes in the mean residue ellipticity and protein spatial structure were registered (Table 3). This effect was likely caused by the influence of Qi binding on HSA’s sulfhydryl groups and their reactivity. However, it is not the same as confirming the modification of the HSA’s secondary structure. To confirm this phenomenon, an analysis of the effect of the interaction of Qi with HSA on the HSA’s secondary structure elements was necessary. As mentioned previously, the only free sulfhydryl (thiol) group in the HSA molecule, which is largely responsible for HSA antioxidant activity, is the amino acid residue Cys-34 [16,17,19,47]. To carry out the secondary structure analysis, the secondary structure estimation program with Young’s reference model was used, and the formation of α-helical conformation (the increase in % α-helix value) and a reduction in the β-sheet content were registered (Table 4). The conformation of a protein can be easily changed due to the binding of ligands, and minor as well as major modifications of the secondary structure of HSA can contribute to the change in the availability of functional groups of side chains of different amino acid residues. Changes in the location, chemical activity, and accessibility may also be related to the Cys-34 thiol group. As Jovanović et al. in their study showed, the presence of fatty acids in the environment significantly impacts the level of available sulfhydryl groups in albumin solution [48]. There is also some evidence to suggest that an increase in both the level of the free thiol groups (in HSA) and chemical reactivity can be observed in the case of clozapine, ziprasidone [49], enterolactone, and enterodiol [50]. 

Under the influence of various ligands, there are possible significant changes in the secondary structure of HSA and slight modifications/interactions, which may also be imperceptible. The above statement is based on numerous literature sources [51,52,53,54,55]. As Owczarzy et al. showed, 5-alkyl-12(H)-quino[3,4-b][1,4]benzothiazinium probably binds to the HSA in Sudlow’s sites I and II. Despite this, changes in the percentage of α-helix below 0.5% were defined as “not changed significantly” [52]. In turn, in the case of commonly used drugs, such as furosemide, changes in the percentage of α-helix in the HSA’s secondary structure can be bigger (at the level of 1–2%). They were defined by Zaidi et al. as “slight structural changes” [51]. Regardless of the presence of Qi, HSA α-helix remains the dominant secondary structure (Table 4). An observed increase (at the level of 1%) in the percentage of α-helix and a decrease in the protein percentage of β-sheet confirm the influence of Qi on HSA’s secondary structure. Due to the binding of various ligands by HSA, such as curcumin or diacetylcurcumin, slight changes in the proportion of α-helix versus β-sheet are possible [56]. The demonstrated domination of the α-helix in HSA’s second-order structure is consistent with the literature data [45,46,57]. The molar ratio of the ligand to HSA is very important in the analysis of the effect of the ligand on HSA’s secondary structure. In the work of Zhu et al. at the molar ratio of atrazine:HSA (atrazine was a triazine herbicide) 1:1 and 1:2, the percentage content of the α-helix of HSA was 30.3% and 27.3%, respectively (initial content 32.5%). The authors described the changes as “change in the conformation of HSA” [53]. In this work, the molar ratio was high (Qi:HSA 6.67:1), while the change in the percentage content of the secondary structure elements of HSA was slight (at the level of 1–2%). This suggests that Qi may probably have little effect/influence on the HSA’s secondary structure. At the same time, this effect/influence is too small to be unambiguously called “change in the conformation of HSA.” This conclusion is consistent with the results of Yuan et al. [54]. They analyzed the binding of two ligands: (−)-epigallocatechin-3-gallate (EGCG) and 5-fluorouracil (FU) with HSA. In the case of both drugs, a wide range of concentration ratios (drug:HSA molar ratio) and a wavelength range similar to that used in this study (in the course of CD measurements) were taken into account. They suggested that a change in the percentage content of the α-helix in HSA of less than 10% means no change in The HSA’s secondary structure [54]. Even small drug interactions on the HSA’s secondary structure may be associated with changes or modifications in the exposure of individual amino acid residues in the HSA molecule. Using CD, Liu et al. observed the decrease in BSA’s α-helix percentage under the influence of cinchona alkaloids, and they explained the fact that protein conformational changes are associated with the exposure of hydrophobic amino acid residues. A decrease in the percentage of the content of α-helix in BSA under the influence of interactions with ligand might suggest the migration of ligand (Qi) inside the BSA structure. It is probably associated with the disturbance of the structure of hydrogen bonds in the protein and modification of the protein’s conformation [35,58]. The slight increase in the percentage of content of α-helix in HSA observed in this study due to the interaction with Qi may probably mean that the amount or stability of hydrogen bonds at the level of the HSA’s secondary structure (Table 4) slightly increases.

### 2.2. The Analysis of Qi and HSA’s Antioxidant Activity

**Figure 3:**
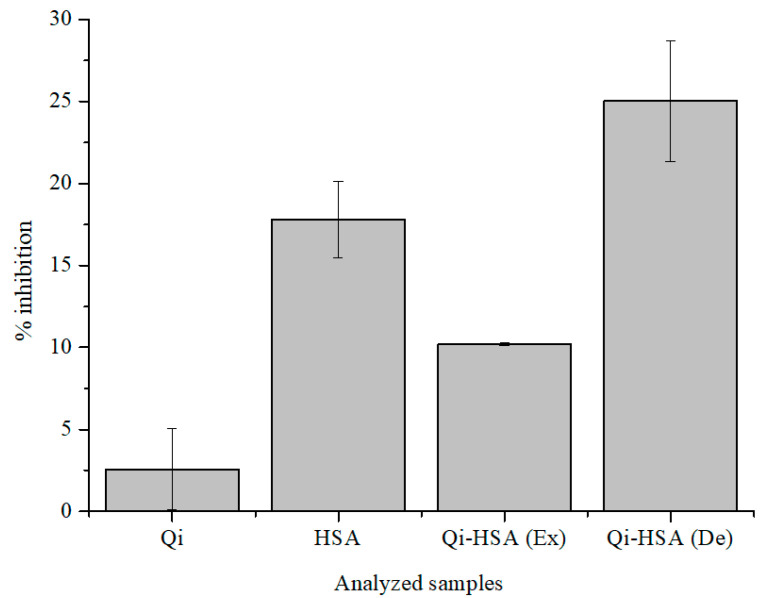
The value of % inhibition of Qi ([Qi] = 7 × 10^−5^ M), HSA ([HSA] = 2.8 × 10^−4^ M) and Qi-HSA_complex_ (1:4 molar ratio; [Qi] = 7 × 10^−5^ M; [HSA] = 2.8 × 10^−4^ M); Ex: expected, De: designated (DPPH assay); pH = 7.4.

Figure 3 presents the value of Qi and HSA, separately and in the mixture (Qi-HSA_complex_), as well as the percentage of inhibition (% inhibition) (DPPH assay).

It has been shown that Qi shows antioxidant activity under denaturing conditions and is a weaker free radical scavenger than HSA (Figure 3; DPPH assay). Qi has the ability to modulate the antioxidant activity of HSA, and the % inhibition value for the HSA solution is 17.79 ± 2.31% and for the Qi-HSA_complex_ mixture is 25.02 ± 3.69% (Figure 3). The designated antioxidant activity value of the Qi-HSA_complex_ mixture (De) was almost two times larger than the expected value (Ex). The expected value of the antioxidant activity of the Qi-HSA_complex_ mixture was calculated, using the mathematical method (the weighted average of the antioxidant activity of HSA and Qi in pure form, taking into account the volume ratio that was used in making the mixture), based on the work of Guimarães et al. [59,60]. They analyzed the different types of interactions in the field of antioxidant activity between three types of herbs. The storage times of collected and prepared herbal products were: 0, 30, 60, or 120 days. Next, on the basis of two-component herbal mixtures, infusions or decoctions have been prepared. An analysis of the antioxidant activity of herbal mixtures were made with the use of reducing the power analysis, DPPH, TBRS, and β-carotene bleaching in the presence of linoleic acid radicals assays. From this work’s point of view, the most important was the analysis of the type of interaction between herbal mixture ingredients. Guimarães et al., in order to calculate the predicted values of antioxidant activity parameters for herbal mixtures (“the theoretical values”), used results of antioxidant activity analysis of individual herbs [59,60].

Table 5 presents the value of Qi and HSA, separately and in the mixture (Qi-HSA_complex_), as well as the percentage of inhibition (% inhibition) (ABTS assay). The amount of vitamin C concentration that expresses the value of the ascorbic acid equivalent antioxidant capacity (AAEAC) ([AA] = 10^−6^ M) was calculated based on the ABTS assay. Table 6 presents the total amount of AAEAC antioxidant activity (ABTS assay), while in Table 7, the ascorbic acid equivalent reduction potential (AAERP) values were determined experimentally (FRAP assay).

Due to the fact that many chemicals can interact with HSA, these substances may affect the antioxidant potential of HSA as well as the HSA–ligand system, e.g., stilbenoids [61], resveratrol [62], or different phenolic acids, as well as gallic acid [63,64]. The antioxidant potential of HSA changes during the course of diabetes in the presence of flavone or glucose [65]. Glyco-oxidative modification of HSA can affect the process of inhibiting the oxidation reaction, especially LDL (low-density lipoprotein) particles [66]. There are cases where ligand binding by HSA has a small effect (or no effect) on the protein’s antioxidant activity, such as the binding of dabrafenib to HSA. It can be connected with the location of the HSA binding site, where amino acid residues are not involved in the free radical scavenging process. Dabrafenib does not interact with Cys-34, which means that the antioxidant activity of this amino acid residue remains unchanged [67]. Bae et al. showed that HSA is responsible for the increased stability of (–)-epigallocatechin gallate (EGCg). It is connected with the antioxidant activity of the Cys-34 thiol group reactivity. Simultaneously, the incubation process of BSA with EGCg has become the cause of the significant increase in the antioxidant activity of the protein. It has been demonstrated with the help of FRAP and the oxygen radical antioxidant capacity (ORAC) assays [68,69].

Based on the data collected in Table 5 and Table 6 and Figure 3, we can conclude that the antioxidant activity of Qi is very low and increases over time. According to the comparison of the data in Table 7, the reduction potential of Qi is also noticeable. The influence of Qi on the level of free radicals in the human body is significant, and it confirms that Qi reduces the activity of antioxidant enzymatic mechanisms and slows the reduction of inflammation [70,71]. It is noteworthy that Qi as a chemical compound has in its structure numerous considerable reductants (–OH and α, β carbonyl moiety), which determine antioxidant activity [72,73,74]. In order to study the antioxidant and reduction potential of analyzed samples (HSA, Qi), ABTS, and FRAP assays were used. Compared to Qi, HSA has a higher free radical scavenging ability, and the antioxidant and reduction potentials were also observed for the mixture in Qi-HSA_complex_ (Table 6 and Table 7). The antioxidant potential of Qi increases with time, in the range between 5 and 60 min from the beginning of the reaction (Table 5). The highest value of AAEAC calculated for HSA was recorded for 60 min, as well as in the presence of Qi (Qi-HSA_complex_; Table 6). This phenomenon has not been observed using the FRAP assay—the obtained AAERP values for both samples were very similar (Table 7). The lowest values of AAERP, regardless of sample incubation time, were observed for Qi.

Based on the data presented in Table 7, the analysis of the interaction between Qi and HSA (FRAP assay) was characterized. The results are presented in Table 8.

Based on the data collected in Table 8, it can be concluded that the interaction between Qi and HSA is characterized by a synergistic nature (Table 8). It means that Qi shows the ability to enhance the HSA antioxidant and reduction potential, irrespective of the samples’ incubation time (from 0 to 60 min). Ex and De values were calculated as mentioned above. The correlation between AAERP and the influence of time was calculated based on Pearson’s correlation coefficient (Table 9).

As shown in Table 9, there is a strong negative correlation between the passage of time and AAERP calculated for Qi. For HSA, the correlation between AAERP and the passage of time is moderately negative. There is also a low negative correlation between the passage of time and AAERP, calculated for Qi-HSA_complex_ (Table 9).

According to the data presented in Table 9, only for Qi, the passage of time has a very high correlation with the change of the antioxidant and reduction potential. With the passage of time in the range from 0 to 60 min, Qi more effectively removes free radicals from the environment; however, for the same time range, its reduction potential decreases. For HSA as well as in the Qi-HSA_complex_, it has been observed that the correlation was (sequentially) low and moderate, and the presence of Qi in the complex with HSA is responsible for the changes in correlation between the passage of time and change in the HSA antioxidant and reduction potential. Pearson’s correlation in the analysis of free radicals was used very frequently. The main goal of Basu et al. [76] and Suleiman et al. was the analysis of the correlation between the total phenolic (TPC) and flavonoid (TFC) contents and antioxidant capacities [76,77]. There are a few works that analyze the correlation between the passage of time and changes in antioxidant potential [78]. The present work is a kind of novelty. A similar analysis of the HSA antioxidant activity modifications was made by Li et al. [79]. They analyzed the interaction between DPPH and HSA, as well as an influence on DPPH-HSA interaction with several antioxidants, such as AA, glutathione, melatonin, or astaxanthin. Most importantly, they showed that these antioxidants can modify the bond between the protein and free radical formation [79]. This in turn provides proof that Qi has a similar impact on the interaction between HSA and other free radicals.

## 3. Materials and Methods

### 3.1. Reagents and Chemicals

**Figure 4:**
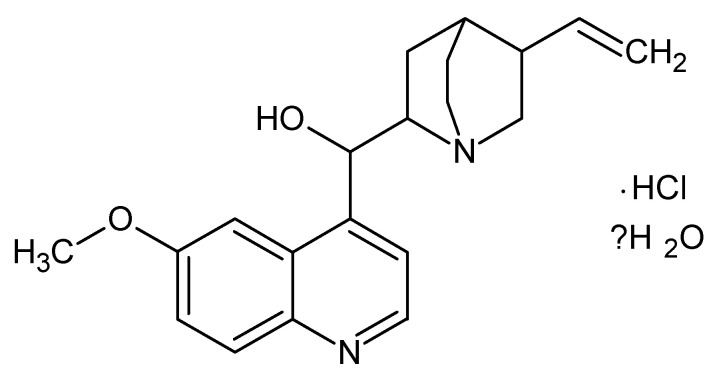
The structural formula of Qi (ChemSketch 12.1.0.31258).

HSA, factor V, was purchased from MP Biomedicals. Dipotassium hydrogen phosphate pure p.a., potassium persulfate (K_2_S_2_O_8_), hydrochloric acid standard solution 1 M (HCl), and AA (C_6_H_8_O_6_) were obtained from Chempur, while sodium dihydrogen phosphate and iron (III) chloride (FeCl_3_) were purchased from EuroChem BGD. Quinine monohydrochloride dihydrate (Figure 4), 2,2′-Azino-bis(3-ethylbenzothiazoline-6-sulfonic acid) diammonium salt (ABTS), and 6-hydroxy-2,5,7,8-tetramethylchroman-2-carboxylic acid (TPTZ) were purchased from Sigma Aldrich. All chemicals were of analytical grade and used without further purification.

### 3.2. Methods

#### 3.2.1. Emission and Absorption Spectra Measurements

Using JASCO FP-6500 Spectrofluorometer, emission fluorescent spectra of 3 × 10^−6^ M HSA in phosphate buffer (10 × 10^−3^ M, pH 7.4), in both the absence and presence of Qi at 1 × 10^−3^ M concentration, were obtained (Qi:HSA molar ratio from 0.33:1 to 6.67:1). *λ_ex_* was 285 nm. The measurements were made at 293 K, in quartz cuvettes at an optical path length of 10 mm (10 × 10 × 40 mm). The accuracy of the wavelength measurement was ±1.5 nm. The phosphate buffer spectrum was subtracted from each of the obtained spectra.

The phenomenon of the absorption of light at excitation (*λ_ex_* 285 nm) and emission (*λ_em_* 333 nm) wavelengths (inner filter effect, IFE) were observed. Because of this, a correction of the Qi-HSA_complex_ system fluorescence intensity was required. For this purpose, the absorbance measurements at the excitation (*λ_ex_* 285 nm) and emission (*λ_em_* 333 nm) wavelengths were taken, and JASCO V-530 Spectrophotometer was used. The IFE correction Equation (1) was used [80]:

(1) $$F_{cor}=F_{obs\;}\times e^{\frac{A_{ex}+A_{em}}{2}}$$

where *F_cor_* is the corrected fluorescence and *F_obs_* is the observed fluorescence (in both cases after the subtraction of the scattering spectrum of the solvent); *A_ex_* is the absorbance at the excitation wavelength (*λ_ex_* 285 nm); *A_em_* is the absorbance at the emission wavelength (*λ_em_* 333 nm).

Due to the necessity to eliminate the influence of absorption by Qi on the obtained results, the excitation wavelength (*λ_ex_*) was selected to be 285 nm.

The Klotz Equation (2) was used to determine the association constants (*K_a_*) in the ligand–protein (Qi-HSA) complex [81]:

(2) $$\frac{1}{r}=\frac{1}{nK_{a}L_{f}}+\frac{1}{n}$$

where *r* is the number of ligand moles bound to 1 mole of the protein; *r* = $\frac{L_{b}}{\left[HSA\right]}$, where *L_b_* is the bound ligand concentration (M) and [HSA] is the total protein concentration (M); n is the number of binding sites; *K_a_* is the association constant (M); *L_f_* is a free ligand concentration (M).

#### 3.2.2. Circular Dichroism (CD) Measurements

The CD spectra of 3.0 × 10^−6^ M HSA in phosphate buffer (pH 7.4) in the absence and presence of 1.0 × 10^−3^ M Qi were recorded using JASCO J-1500 Spectropolarimeter. HSA was mixed with Qi in the protein:drug molar ratio 1:6.67. The measurements were made at 298 K by a thermostated Peltier cell holder with an accuracy of ±0.05 K, in quartz cuvettes with an optical path length of 1 mm. The accuracy of the wavelength measurement was ±0.1 nm, and the wavelength repeatability was ±0.05 nm. The spectrum CD of the phosphate buffer was subtracted from each of the obtained spectra. Smoothing of the CD spectrum was performed with a Savitzky–Golay filter, a second order polynomial, and 11 data points.

The mean residue ellipticity [Θ]_mrw_ was calculated using Equation (3) [41,82]:

(3) $$\left[\Theta {]}_{\mathrm{mrw}}=\frac{\mathrm{MRW}\;\times \;\Theta \;}{10\times \;d\;\times \;m\;}\;[\deg\;\times \;{\mathrm{cm}}^{2}\times \;{\mathrm{dmol}}^{-1}\right]$$

where Θ is the observed ellipticity for a given wavelength (deg); MRW is the mean residue weight (MRW_HSA_ = 113.7 Da); m is the concentration of HSA (g/cm^3^); d is the optical path length (cm).

#### 3.2.3. Interaction Ligand–Protein Analysis

A JASCO V-730 spectrophotometer was used for the spectroscopic analysis of interactions between Qi and HSA. Absorption measurements were made in the wavelength range from 300 to 370 nm. The following solutions were tested: Qi (7 × 10^−5^ M), HSA (7 × 10^−5^ M, 1.4 × 10^−4^ M, 2.8 × 10^−4^ M), and Qi-HSA_complex_ (Qi:HSA molar ratio 1:1; 1:2; 1:4). Qi_calc_ = Qi-HSA_complex_ minus HSA). All tested solutions were prepared in phosphate buffer (pH 7.4).

#### 3.2.4. Reduction and Antioxidant Potential Analysis

DPPH, ABTS, and FRAP assay measurements were conducted using a JASCO V-730 spectrophotometer. In DPPH, ABTS, and FRAP assays, the value of absorbance was analyzed for 515, 734, and 593 nm wavelengths, respectively. The optical path length was 10 mm (T 293 K). Measurements were made in the following intervals: 0, 10, 20, 30, and 60 min (FRAP assay); 5, 15, 30, 45, and 60 min (ABTS assay), or 5 min (DPPH assay) after initiating the radical reaction. All samples were prepared in phosphate buffer (pH = 7.4) and stored in the dark.

DPPH assay: DPPH (2,2-diphenylo-1-picrylhydrazyl) is a commonly known and used model free radical. It has an unpaired electron on the valence shell of the nitrogen atom. Under the influence of antioxidants, the ethanolic solution of DPPH turns from violet to yellow, and the absorbance drops at a wavelength of 515 nm [83,84]. HSA (2.8 × 10^−4^ M), Qi (7 × 10^−5^ M), and Qi-HSA_complex_ (molar ratio 1:4) solutions were mixed with the DPPH solution (1 × 10^−4^ M) in (*v*/*v*) volume ratio 1:1.

ABTS assay: ABTS (2,2′-azino-bis(3-ethylbenzothiazoline-6-sulfonic acid) is a chemical compound that generates a radical cation. The ABTS assay is one of the easiest and most reliable ways of antioxidant activity analysis of substances such as albumin, which denatures in methanol solution and at a low pH. An ABTS assay was used based on the modified protocols of Re et al. and Biskup et al. [85,86]. A working reagent solution was prepared by mixing 7 mL ABTS solution at 5 × 10^−3^ M concentration and potassium persulfate at 1.74 × 10^−3^ M concentration in phosphate buffer (pH 7.4). The mixture of these two substances was incubated for 16 h and then diluted with a solvent until the absorbance reached 1. The Qi-HSA_complex_ solution was prepared by mixing 6.67 × 10^−5^ M Qi and 1 × 10^−5^ M HSA solution at 1:1 (*v*/*v*) volume ratio. A working reagent solution (ABTS reagent) was mixed with 6.67 × 10^−5^ M Qi, 1 × 10^−5^ M HSA, and Qi-HSA_complex_ solution at 1:1 (ABTS reagent/Qi or HSA or Qi-HSA_complex_; *v*/*v*) volume ratio.

In the presence of an antioxidant, the color of the ABTS solution changes from dark green to colorless. Due to the use of ethanol or methanol as a DPPH solvent in order to prevent HSA denaturation, we decided not to use DPPH as a model-free radical interacting with HSA [87,88].

The results of spectrophotometric measurements (DPPH and ABTS assay) were converted into the value of % inhibition using Equation (4) [89,90]:

(4) $$\%\;inhibition=\left(\frac{A_{0}-A_{1}}{A_{0}}\right)\times 100\%$$

where *A*_0_ is the absorbance of ABTS or DPPH (at *λ* 734 or 515 nm) in the absence of the tested substance; *A*_1_ is the absorbance of ABTS or DPPH (at *λ* 734 or 515 nm) in the presence of the tested substance.

Calibration curves for AA were determined. The % inhibition of the tested substances was converted to AA concentrations in the range 0.54 × 10^−6^ M–69.83 × 10^−6^ M, and the AAEAC expressed in 10^−6^ M of the AA solution was obtained.

FRAP assay: Although the FRAP assay is not recommended as a method of albumin antioxidant activity measurements, it is a very promising method of measuring antioxidant reduction potential. A FRAP assay was used based on Benzie et al.’s modified protocol [91]. A working reagent solution was prepared by mixing in volume ratio (*v*/*v*/*v*) 1:1:10 TPTZ (2,4,6-Tri(2-pyridyl)-s-triazine) (0.01 M) in hydrochloric acid (0.04 M), water solution of iron chloride (III) (0.02 M), and acetate buffer (pH 3.6). The antioxidant activity of 9.3 × 10^−5^ M Qi, 6.3 × 10^−4^ M HSA and Qi-HSA_complex_ solution at a 1:7.5 (*v*/*v*) volume ratio with a FRAP reagent were mixed. In the presence of a substance with a reducing potential, a colorless TPTZ complex with Fe^3+^ ions changed to navy blue.

Calibration curves for AA were determined. The absorbance of tested substances was converted to an AA concentration in the range 22 × 10^−6^ M–363 × 10^−6^ M, and the AAERP expressed in 10^−3^ M of the AA solution was obtained.

### 3.3. Statistics

The two (minimum) replicates of the results were expressed as arithmetic mean and relative standard deviation (mean ± SD). Using OriginPro software version 8.5 SR1 (Northampton, MA, USA), Statistica (data analysis software system), version 13; TIBCO Software Inc. 2017 (Palo Alto, CA, USA), Microsoft Excel 2013 (Redmond, Washington, USA), and Spectra Manager Version 2.13.00 2002–2015 JASCO Corporation (Tokyo, Japan) by fitting the experimental data to the appropriate equations, the calibration curves as well as figures and tables, were analyzed. To analyze the correlation between the time and changes in the antioxidant and reduction potential, Pearson’s correlation was used [75].

## 4. Conclusions

The results presented in this study allowed for conclusions that Qi influences the antioxidant and reduction potential of HSA. This phenomenon means that Qi increases the ability of the tested protein to remove free radicals from the reaction environment and decreases the time in which HSA’s antioxidant activity is the highest. The interaction between Qi and HSA has a synergistic effect. Qi is a very weak antioxidant but it reacts with various free radicals. The association constant (K_a_) of Qi and HSA has a very low value, which means that this alkaloid has a low affinity for the analyzed protein.

The presented method of analyzing modulation shows the effect of drugs on endogenous antioxidant mechanisms, which can afford multiple drugs tested in the future and provide precious knowledge. The authors aimed to obtain reliable results regarding the practical application of the studied drug.

## Figures and Tables

**Table 1:** The values of mean absorbance for “Qi” and “Qi_calc_ = Qi-HSA_complex_ − HSA” at *λ_max_* 331 nm in Qi-HSA_complex_ 1:1, 1:2, and 1:4 molar ratio; [Qi] 7 × 10^−5^ M, [HSA] 7 × 10^−5^ M, 1.4 × 10^−4^ M, 2.8 × 10^−4^ M; all tested solutions were prepared in phosphate buffer (pH 7.4).

	1:1	1:2	1:4
**Qi measured (Qi)**	0.413 ± 0.012		
**Qi calculated (Qicalc)**	0.427 ± 0.011	0.399 ± 0.005	0.342 ± 0.000

**Table 2:** The association constant (*K_a_*) and the number of molecules bound to protein (*n*) in Qi-HSA system; Qi:HSA molar ratio from 0.67:1 to 6.67:1; [HSA] 3 × 10^−6^ M; [Qi] 1 × 10^−3^ M; pH = 7.4.

System	*K_a_* [M^−1^] ± SD * × 10^4^	*n* ± SD *
**Qi-HSA_complex_**	0.952 ± 0.178	1.336 ± 0.274

* Standard deviation.

**Table 3:** The CD spectra and the values of mean residue ellipticity [Θ_mrw_] for HSA, in both the absence and presence of Qi (Qi:HSA 6.67:1 molar ratio); [HSA] 3 × 10^−6^ M; [Qi] 1 × 10^−3^ M; pH = 7.4.

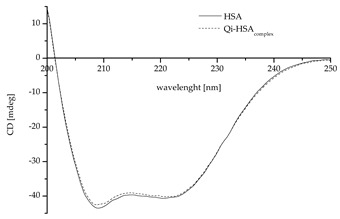		
System	*λ_min_* [nm]	[Θ]_mrw_ ± SD * [deg · cm^2^ · dmol^−1^]
**HSA**	209.0	−24,774.26 ± 20.19
	220.8	−23,180.92 ± 19.47
**Qi-HSA_complex_**	209.0	−24,214.71 ± 21.24
	220.8	−22,925.82 ± 12.78

* Standard deviation.

**Table 4:** The percentage (%) content of the secondary structure elements of HSA, in both the presence and absence of Qi (Qi:HSA 6.67:1 molar ratio); Young’s reference model.

System	% α-Helix ± SD *	% β-Sheet ± SD *	% Turn ± SD *	% Other ± SD *
**HSA**	35.10 ± 0.28	12.25 ± 0.35	21.05 ± 0.35	31.60 ± 0.28
**Qi-HSA_complex_**	36.25 ± 0.07	10.25 ± 0.07	21.50 ± 0.14	32.00 ± 0.01

* Standard deviation.

**Table 5:** The value of % inhibition of Qi ([Qi] = 6.67 × 10^−5^ M), HSA ([HSA] = 1 × 10^−5^ M), and Qi-HSA_complex_ (1:6.67 molar ratio; [Qi] = 6.67 × 10^−5^ M; [HSA] = 6.67 × 10^−5^ M; ABTS assay); pH = 7.4).

% Inhibition ± SD *					
Time [min]	5	15	30	45	60
Qi	1.38 ± 0.75	1.09 ± 0.89	1.69 ± 1.86	2.45 ± 2.26	3.48 ± 2.62
HSA	26.75 ± 0.55	49.11 ± 0.24	57.45 ± 0.24	62.81 ± 0.32	67.31 ± 1.07
Qi-HSA_complex_	19.28 ± 1.10	33.01 ± 1.62	39.36 ± 1.56	44.07 ± 1.25	47.71 ± 0.53

* Standard deviation.

**Table 6:** The total AAEAC antioxidant capacity (ABTS assay); pH = 7.4).

AAEAC ± SD * [10^−6^ M AA]					
Time [min]	5	15	30	45	60
Qi	0.96 ± 0.61	ND **	ND **	ND **	ND **
HSA	21.63 ± 0.44	40.07 ± 0.21	49.01 ± 0.22	54.40 ± 0.30	57.05 ± 0.99
Qi-HSA_complex_	15.55 ± 0.90	25.95 ± 1.42	32.66 ± 1.41	36.83 ± 1.17	38.95 ± 0.49

* Standard deviation; ** No data; indeterminate values due to too low Qi antioxidant activity (Table 5).

**Table 7:** The total AAERP antioxidant capacity (FRAP assay); pH = 7.4).

AAERP ± SD * [10^−6^ M AA]					
Time [min]	0	10	20	30	60
Qi	57.70 ± 2.31	52.66 ± 1.79	46.23 ± 2.02	43.59 ± 1.83	38. 50 ± 2.32
HSA	57.76 ± 2.94	61.74 ± 3.52	56.07 ± 5.12	56.47 ± 6.01	54.72 ± 8.45
Qi-HSA_complex_	60.16 ± 2.25	64.72 ± 4.51	61.84 ± 5.38	61. 49 ± 6.70	59.94 ± 8.84

* Standard deviation.

**Table 8:** Expected versus designated values of AAERP.

Assay	Time [min]		Qi-HSA_complex_ ± SD *	Assay	Time [min]		Qi-HSA_complex_ ± SD *
ABTS	5	Ex	11.30 ± 0.09	FRAP	0	Ex	57.73 ± 2.57
		De	15.55 ± 0.90			De	60.16 ± 2.25
		E	s			E	a
	15	Ex	20.04 ± 0.11 **		10	Ex	57.36 ± 2.43
		De	25.95 ± 1.42			De	64.72 ± 4.51
		E	s			E	s
	30	Ex	24.60 ± 0.15 **		20	Ex	51.15 ± 3.45
		De	32.66 ± 1.41			De	61.84 ± 5.38
		E	s			E	s
	45	Ex	27.24 ± 0.09 **		30	Ex	50.03 ± 3.83
		De	36.83 ± 1.17			De	61.49 ± 6.70
		E	s			E	s
	60	Ex	28.67 ± 0.25 **		60	Ex	46.61 ± 5.16
		De	38.95 ± 0.49			De	59.94 ± 8.84
		E	s			E	s

Ex—expected, De—designated, E—effect; additive effect (a)—theoretical and experimental values reveal differences lower than 5%; synergistic effect (s)—experimental values are more than 5% higher for AAEAC or AAERP when compared with theoretical values; antagonistic effect (an)—experimental values are more than 5% smaller for AAEAC or AAERP when compared with theoretical values [59,60]; * Standard deviation; ** It was assumed that the AAEAC value is zero in samples where it was impossible to determine the AAEAC value (Table 5 and Table 6).

**Table 9:** Pearson’s r correlation coefficient—FRAP assay.

Assay	Sample	Linear Regression Equation	R^2^	Pearson’s r Correlation Coefficient	The Nature of Correlation *
FRAP	Qi	y = −0.0003x + 0.0552	0.8983	−0.9478	Very high
	HSA	y = −0.00008x + 0.0592	0.4523	−0.6725	Moderate
	Qi-HSA_complex_	y = −0.00003x + 0.0624	0.1605	−0.4006	Low

* Very high correlation: size of correlation between 0.9 and 1.0 (−0.9 and −1.0); high correlation: size of correlation between 0.7 and 0.9 (−0.7 and −0.9); moderate correlation: size of correlation between 0.5 and 0.7 (−0.5 and −0.7); low correlation: size of correlation between 0.3 and 0.5 (−0.3 and −0.5); negligible correlation: size of correlation between 0.0 and 0.3 (−0.0 and −0.3) [75].

## Data Availability

The data presented in this study are available upon request from the corresponding author.

## References

[1] Achan J., Talisuna A.O., Erhart A., Yeka A., Tibenderana J.K., Baliraine F.N., Rosenthal P.J., D’Alessandro U. (2011). Quinine, an old anti-malarial rug in a modern world: Role in the treatment of malaria. Malar. J..

[2] Jones R.A., Panda S.S., Hall C.D. (2015). Quinine conjugates and quinine analogues as potential antimalarial agents. Eur. J. Med. Chem..

[3] Permin H., Norn S., Kruse E., Kruse P.R. (2016). On the history of Cinchona bark in the treatment of Malaria. Dan. Medicinhist. Arbog..

[4] Rogerson S.J. (2017). Management of malaria in pregnancy. Indian J. Med. Res..

[5] Lalloo D.G., Shingadia D., Bell D.J., Beeching N.J., Whitty C.J.M., Chiodini P.L., PHE Advisory Committee on Malaria Prevention in UK Travellers (2016). UK malaria treatment guidelines 2016. J. Infect..

[6] Kehrer J.P., Klotz L.O. (2015). Free radicals and related reactive species as mediators of tissue injury and disease: Implications for Health. Crit. Rev. Toxicol..

[7] Greve B., Lehman L.G., Lell B., Luckner D., Schmidt-Ott R., Kremsner P.G. (1999). High oxygen radical production is associat-ed with fast parasite clearance in children with Plasmodium falciparum malaria. J. Infect. Dis..

[8] Kharazmi A., Jepsen S., Andersen B.J. (1987). Generation of reactive oxygen radicals by human phagocytic cells activated by Plasmodium falciparum. Scand. J. Immunol..

[9] Jones D.P. (2008). Radical-free biology of oxidative stress. Am. J. Physiol. Cell Physiol..

[10] Alkadi H. (2020). A Review on Free Radicals and Antioxidants. Infect. Disord. Drug Targets.

[11] Arques S. (2018). Albumine sérique et maladies cardiovasculaires: Une revue approfondie de la littérature [Serum albumin and cardiovascular diseases: A comprehensive review of the literature. Ann. Cardiol. Angeiol..

[12] Wanwimolruk S., Denton J.R. (1992). Plasma protein binding of quinine: Binding to human serum albumin, alpha 1-acid gly-coprotein and plasma from patients with malaria. J. Pharm. Pharmacol..

[13] Olszowy-Tomczyk M. (2020). Synergistic, antagonistic and additive antioxidant effects in the binary mixtures. Phytochem. Rev..

[14] Lushchak V.I. (2014). Free radicals, reactive oxygen species, oxidative stress and its classification. Chem. Biol. Interact..

[15] Gebicki J.M. (2016). Oxidative stress, free radicals and protein peroxides. Arch. Biochem. Biophys..

[16] Soriani M., Pietraforte D., Minetti M. (1994). Antioxidant potential of anaerobic human plasma: Role of serum albumin and thiols as scavengers of carbon radicals. Arch. Biochem. Biophys..

[17] Anraku M., Chuang V.T., Maruyama T., Otagiri M. (2013). Redox properties of serum albumin. Biochim. Biophys. Acta.

[18] Fanali G., di Masi A., Trezza V., Marino M., Fasano M., Ascenzi P. (2012). Human serum albumin: From bench to bedside. Mol. Aspects Med..

[19] Plantier J.L., Duretz V., Devos V., Urbain R., Jorieux S. (2016). Comparison of antioxidant properties of different therapeutic albumin preparations. Biologicals.

[20] Maciążek-Jurczyk M., Sułkowska A. (2015). Spectroscopic analysis of the impact of oxidative stress on the structure of human serum albumin (HSA) in terms of its binding properties. Spectrochim. Acta A Mol Biomol. Spectrosc..

[21] Li X., Chen D., Wang G., Lu Y. (2013). Study of interaction between human serum albumin and three antioxidants: Ascorbic acid, α-tocopherol, and proanthocyanidins. Eur. J. Med. Chem..

[22] Lee H., Cha M.K., Kim I.H. (2000). Activation of thiol-dependent antioxidant activity of human serum albumin by alkaline pH is due to the B-like conformational change. Arch. Biochem. Biophys..

[23] Vieira J.L.F., Borges L.M.G., Nascimento M.T.S., de Gomes A.L.S. (2008). Quinine levels in patients with uncomplicated fal-ciparum malaria in the Amazon region of Brazil. Quinine levels in patients with uncomplicated falciparum malaria in the Ama-zon region of Brazil. Braz. J. Infect. Dis..

[24] Valko M., Jomova K., Rhodes C.J., Kuča K., Musílek K. (2016). Redox- and non-redox-metal-induced formation of free radicals and their role in human disease. Arch. Toxicol..

[25] Al-Harthi S., Lachowicz J.I., Nowakowski M.E., Jaremko M., Jaremko Ł. (2019). Towards the functional high-resolution co-ordination chemistry of blood plasma human serum albumin. J. Inorg. Biochem..

[26] McCloskey K.L., Garriott J.C., Roberts S.M. (1978). Quinine Concentrations in Blood Following the Consumption of Gin and Tonic Preparations in a Social Setting. J. Anal. Toxicol..

[27] Silamut K., White N.J., Looareesuwan S., Warrell D.A. (1985). Binding of quinine to plasma proteins in falciparum malaria. Am. J. Trop. Med. Hyg..

[28] Dyer J.R., Davis T.M., Giele C., Annus T., Garcia-Webb P., Robson J. (1994). The pharmacokinetics and pharmacodynamics of quinine in the diabetic and non-diabetic elderly. Br. J. Clin. Pharmacol..

[29] Pukrittayakamee S., Wanwimolruk S., Stepniewska K., Jantra A., Huyakorn S., Looareesuwan S., White N.J. (2003). Quinine pharmacokinetic-pharmacodynamic relationships in uncomplicated falciparum malaria. Antimicrob. Agents Chemother..

[30] Ren G., Sun H., Guo J., Fan J., Li G., Xu S. (2019). Molecular mechanism of the interaction between resveratrol and trypsin via spectroscopy and molecular docking. Food Funct..

[31] Suhartono E., Thalib I., Aflanie I., Noor Z., Idroes R. (2018). Study of Interaction between Cadmium and Bovine Serum Al-bumin with UV-Vis Spectrocopy Approach. IOP Conf. Ser. Mater. Sci. Eng..

[32] Frostell-Karlsson A., Remaeus A., Roos H., Andersson K., Borg P., Hämäläinen M., Karlsson R. (2000). Biosensor analysis of the interaction between immobilized human serum albumin and drug compounds for prediction of human serum albumin binding levels. J. Med. Chem..

[33] Bougie D.W., Wilker P.R., Aster R.H. (2006). Patients with quinine-induced immune thrombocytopenia have both “drug-dependent” and “drug-specific” antibodies. Blood.

[34] Marszałł M.P., Buciński A. (2010). A protein-coated magnetic beads as a tool for the rapid drug-protein binding study. J. Pharm. Biomed. Anal..

[35] Liu Y., Chen M., Wang S., Lin J., Cai L., Song L. (2014). New insight into the stereoselective interactions of quinine and quinidine, with bovine serum albumin. J. Mol. Recognit..

[36] Day Y.S.N., Myszka D.G. (2003). Characterizing a Drug’s Primary Binding Site on Albumin. J. Pharm. Sci..

[37] Kratochwil N.A., Huber W., Müller F., Kansy M., Gerber P.R. (2002). Predicting plasma protein binding of drugs: A new ap-proach. Biochem. Pharmacol..

[38] Sudlow G., Birkett D.J., Wade D.N. (1975). The characterization of two specific drug binding sites on human serum albumin. Mol. Pharmacol..

[39] Paubel J.P., Niviere P. (1974). Bonding of therapeutic organic molecules with proteins quinine. Eur. J. Med. Chem..

[40] Greenfield N.J. (2006). Using circular dichroism spectra to estimate protein secondary structure. Nat. Protoc..

[41] Venyaminov S.Y., Vassilenko K.S. (1994). Determination of protein tertiary structure class from circular dichroism spectra. Anal. Biochem..

[42] Kelly S.M., Jess T.J., Price N.C. (2005). How to study proteins by circular dichroism. Biochim. Biophys. Acta.

[43] Kelly S.M., Price N.C. (2000). The use of circular dichroism in the investigation of protein structure and function. Curr. Protein Pept. Sci..

[44] Chudzik M., Maciążek-Jurczyk M., Pawełczak B., Sułkowska A. (2016). Spectroscopic Studies on the Molecular Ageing of Se-rum Albumin. Molecules.

[45] Szkudlarek A., Pentak D., Ploch A., Pożycka J., Maciążek-Jurczyk M. (2017). In Vitro Investigation of the Interaction of Tol-butamide and Losartan with Human Serum Albumin in Hyperglycemia States. Molecules.

[46] Maciążek-Jurczyk M., Janas K., Pożycka J., Szkudlarek A., Rogóż W., Owczarzy A., Kulig K. (2020). Human Serum Albumin Aggregation/Fibrillation and its Abilities to Drugs Binding. Molecules.

[47] Bocedi A., Cattani G., Stella L., Massoud R., Ricci G. (2018). Thiol disulfide exchange reactions in human serum albumin: The apparent paradox of the redox transitions of Cys34. FEBS J..

[48] Jovanović V.B., Pavićević I.D., Takić M.M., Penezić-Romanjuk A.Z., Aćimović J.M., Mandić L.M. (2014). The influence of fatty acids on determination of human serum albumin thiol group. Anal. Biochem..

[49] Uzelac T.N., Nikolić-Kokić A.L., Spasić S.D., Mačvanin M.T., Nikolić M.R., Mandić L.M., Jovanović V.B. (2019). Opposite clozapine and ziprasidone effects on the reactivity of plasma albumin SH-group are the consequence of their different binding properties dependent on protein fatty acids content. Chem. Biol. Interact..

[50] Takić M.M., Jovanović V.B., Pavićević I.D., Uzelac T.N., Aćimović J.M., Ristić-Medić D.K., Mandić L.M. (2016). Binding of enterolactone and enterodiol to human serum albumin: Increase of cysteine-34 thiol group reactivity. Food Funct..

[51] Zaidi N., Ahmad E., Rehan M., Rabbani G., Ajmal M.R., Zaidi Y., Subbarao N., Khan R.H. (2013). Biophysical insight into furosemide binding to human serum albumin: A study to unveil its impaired albumin binding in uremia. J. Phys. Chem. B.

[52] Owczarzy A., Zięba A., Pożycka J., Kulig K., Rogóż W., Szkudlarek A., Maciążek-Jurczyk M. (2021). Spectroscopic Studies of Quinobenzothiazine Derivative in Terms of the In Vitro Interaction with Selected Human Plasma Proteins. Part 1. Molecules.

[53] Zhu M., Wang L., Wang Y., Zhou J., Ding J., Li W., Xin Y., Fan S., Wang Z., Wang Y. (2018). Biointeractions of Herbicide Atrazine with Human Serum Albumin: UV-Vis, Fluorescence and Circular Dichroism Approaches. Int. J. Environ. Res. Public Health.

[54] Yuan L., Liu M., Liu G., Li D., Wang Z., Wang B., Han J., Zhang M. (2017). Competitive binding of (-)-epigallocatechin-3-gallate and 5-fluorouracil to human serum albumin: A fluorescence and circular dichroism study. Spectrochim. Acta A Mol. Biomol. Spectrosc..

[55] Duman O., Tunç S., Kancı Bozoğlan B. (2013). Characterization of the binding of metoprolol tartrate and guaifenesin drugs to human serum albumin and human hemoglobin proteins by fluorescence and circular dichroism spectroscopy. J. Fluoresc..

[56] Mohammadi F., Bordbar A.K., Divsalar A., Mohammadi K., Saboury A.A. (2009). Analysis of binding interaction of curcu-min and diacetylcurcumin with human and bovine serum albumin using fluorescence and circular dichroism spectroscopy. Protein J..

[57] Messina P., Prieto G., Dodero V., Ruso J.M., Schulz P., Sarmiento F. (2005). Ultraviolet-circular dichroism spectroscopy and potentiometric study of the interaction between human serum albumin and sodium perfluorooctanoate. Biopolymers.

[58] Liu Y., Chen M., Jiang L., Song L. (2015). Stereoselective interaction of cinchona alkaloid isomers with bovine serum albu-min. Food Chem..

[59] Guimarães R., Barros L., Carvalho A.M., Ferreira I.C. (2011). Infusions and decoctions of mixed herbs used in folk medicine: Synergism in antioxidant potential. Phytother. Res..

[60] Guimarães R., Barreira J.C.M., Barros L., Carvalho A.M., Ferreira I.C.F.R. (2011). Effects of oral dosage forms and storage period in the antioxidant properties of four species used in traditional herbal medicine. Phytother. Res..

[61] Cao H., Jia X., Shi J., Xiao J., Chen X. (2016). Non-covalent interaction between dietary stilbenoids and human serum albu-min: Structure-affinity relationship, and its influence on the stability, free radical scavenging activity and cell uptake of stil-benoids. Food Chem..

[62] Arcanjo N.M.O., Luna C., Madruga M.S., Estévez M. (2018). Antioxidant and pro-oxidant actions of resveratrol on human se-rum albumin in the presence of toxic diabetes metabolites: Glyoxal and methyl-glyoxal. Biochim. Biophys. Acta Gen. Subj..

[63] Cao H., Chen X., Yamamoto K. (2012). Bovine serum albumin significantly improves the DPPH free radical scavenging po-tential of dietary polyphenols and gallic acids. Anticancer Agents Med. Chem..

[64] Zhang Y., Wu S., Qin Y., Liu J., Liu J., Wang Q., Ren F., Zhang H. (2018). Interaction of phenolic acids and their derivatives with human serum albumin: Structure-affinity relationships and effects on antioxidant activity. Food Chem..

[65] Du S., Xie Y., Chen X. (2013). Influence of glucose on the human serum albumin-flavone interaction and their antioxidant activity. Mol. Biosyst..

[66] Sakata N., Moh A., Takebayashi S. (2002). Contribution of superoxide to reduced antioxidant activity of glycoxidative serum albumin. Heart Vessels.

[67] Suo Z., Xiong X., Sun Q., Zhao L., Tang P., Hou Q., Zhang Y., Wu D., Li H. (2018). Investigation on the Interaction of Dabrafenib with Human Serum Albumin Using Combined Experiment and Molecular Dynamics Simulation: Exploring the Binding Mechanism, Esterase-like Activity, and Antioxidant Activity. Mol. Pharm..

[68] Almajano M.P., Delgado M.E., Gordon M.H. (2007). Changes in the antioxidant properties of protein solutions in the presence of epigallocatechin gallate. Food Chem..

[69] Bae M.J., Ishii T., Minoda K., Kawada  Y., Ichikawa T., Mori T., Kamihira M., Nakayama  T. (2009). Albumin stabilizes (-)-epigallocatechin gallate in human serum: Binding capacity and antioxidant property. Mol. Nutr. Food Res..

[70] Adeniyi O.S., Makinde O.V., Friday E.T., Olaleye S.B. (2017). Effects of quinine on gastric ulcer healing in Wistar rats. J. Complement. Integr. Med..

[71] Gbotolorun S.C., Inikori O., Bamisi O.D., Osinubi A.A.A., Okanlawon A.O. (2018). Quinine inhibits ovulation and produces oxidative stress in the ovary of cyclic Sprague-Dawley rats. Afr. Health Sci..

[72] Milugo T.K., Omosa L.K., Ochanda J.O., Owuor B.O., Wamunyokoli F.A., Oyugi J.O., Ochieng J.W. (2013). Antagonistic ef-fect of alkaloids and saponins on bioactivity in the quinine tree (Rauvolfia caffra sond.): Further evidence to support biotechnol-ogy in traditional medicinal plants. BMC Complement. Altern. Med..

[73] Krishnaveni M., Suresh K., Rajasekar M. (2015). Antioxidant and free radical scavenging activity of quinine determined by using different in vitro models. Int. J. Modn. Res. Revs..

[74] Krishnaveni M., Suresh K. (2015). A Study on Protective Effect of Quinine against Lipid Peroxidation and Antioxidants Status in Human Oral Cancer Cell Line. Res. J. Pharm. Biol. Chem. Sci..

[75] Mukaka M.M. (2012). Statistics Corner: A guide to appropriate use of Correlation coefficient in medical research. Malawi Med. J..

[76] Basu P., Maier C. (2016). In vitro Antioxidant Activities and Polyphenol Contents of Seven Commercially Available Fruits. Pharmacogn. Res..

[77] Suleiman M.H.A., Ateeg A.A. (2020). Antimicrobial and Antioxidant Activities of Different Extracts from Different Parts of *Zilla spinosa* (L.) Prantl. Evid. Based Complement. Alternat. Med..

[78] Henríquez C., López-Alarcón C., Gómez M., Lutz M., Speisky H. (2011). Time-dependence of ferric reducing antioxidant power (FRAP) index in Chilean apples and berries. Arch. Latinoam. Nutr..

[79] Li X., Chen D., Wang G., Lu Y. (2015). Probing the interaction of human serum albumin with DPPH in the absence and pres-ence of the eight antioxidants. Spectrochim. Acta A Mol Biomol. Spectrosc..

[80] Kirby E.P., Steiner R.F., Weinryb I. (1971). Fluorescence Instrumentation and Methodology, Chapter 2. Excited States of Proteins and Nucleic Acids.

[81] Klotz I.M., Hunston D.L. (1975). Protein interactions with small molecules. Relationships between stoichiometric binding constants, site binding constants, and empirical binding parameters. J. Biol. Chem..

[82] Sreerama N., Woody R.W. (2000). Estimation of Protein Secondary Structure from Circular Dichroism Spectra: Comparison of CONTIN, SELCON, and CDSSTR Methods with an Expanded Reference. Set. Anal. Biochem..

[83] Blois M.S. (1958). Antioxidant determinations by the use of a stable free radical. Nature.

[84] Tepe B., Sokmen M., Akpulat H.A., Sokmen A. (2005). In vitro antioxidant activities of the methanol extracts of four Heli-chrysum species from Turkey. Food Chem..

[85] Re R., Pellegrini N., Proteggente A., Pannala A., Yang M., Rice-Evans C. (1999). Antioxidant activity applying an improved ABTS radical cation decolorization assay. Free Radic. Biol. Med..

[86] Biskup I., Golonka I., Gamian A., Sroka Z. (2013). Antioxidant activity of selected phenols estimated by ABTS and FRAP methods. Postepy Hig. Med. Dosw..

[87] Rogóż W., Pożycka J., Owczarzy A., Kulig K., Maciążek-Jurczyk M. (2022). Comparison of Losartan and Furosemide Interaction with HSA and Their Influence on HSA Antioxidant Potential. Pharmaceuticals.

[88] Bersuder P., Hole M., Smith G. (1998). Antioxidants from a heated histidine-glucose model system. I: Investigation of the an-tioxidant role of histidine and isolation of antioxidants by high-performance liquid chromatography. J. Amer. Oil Chem. Soc..

[89] Siegei S., Frost P., Porto F. (1960). Effects of Indoleacetic Acid and Other Oxidation Regulators on in Vitro Peroxidation and Experimental Conversion of Eugenol to Lignin. Plant Physiol..

[90] Molyneux P. (2004). The use of the stable free radical diphenlpicrylhydrazyl (DPPH) for estimating antioxidant activity. Songklanakarin J. Sci. Technol..

[91] Benzie I.F., Strain J.J. (1996). The ferric reducing ability of plasma (FRAP) as a measure of “antioxidant power”: The FRAP as-say. Anal. Biochem..

